# Health Care Organizations: Soft Target during COVID-19 Pandemic

**DOI:** 10.1017/S1049023X2100025X

**Published:** 2021-02-24

**Authors:** Harald G. De Cauwer, Francis Somville

**Affiliations:** 1. Department of Neurology, Dimpna Regional Hospital, Geel, Belgium; 2. Faculty of Medicine and Health Sciences, University of Antwerp, Wilrijk, Belgium; 3. Department of Emergency and Traumatology, Dimpna Regional Hospital, Geel, Belgium; 4. Faculty of Medicine, University of Leuven, Leuven, Belgium

**Keywords:** COVID-19, cyberterrorism, hospitals, pandemic, terrorism, COVID-19, coronavirus disease 2019, CTM, counter-terrorism medicine, EU, European Union, FBI, Federal Bureau of Investigation, SARS-CoV-2, severe acute respiratory syndrome coronavirus-2, UK, United Kingdom, UN, United Nations, US, United States

## Abstract

Health care organizations have been challenged by the coronavirus disease 2019 (COVID-19) pandemic for some time, while in January 2020, it was not immediately suspected that it would take such a global expansion. In the past, other studies have already pointed out that health care systems, and more specifically hospitals, can be a so-called “soft target” for terrorist attacks. This report has now examined whether this is also the case in the context of the COVID-19 pandemic.

During the lockdown, hospitals turned out to be the only remaining soft targets for attacks, given that the other classic targets were closed during the lockdown. On the other hand, other important factors have limited the risk of such attacks in hospitals. The main delaying and relative risk-reducing factors were the access control on temperature and wearing a mask, no visits allowed, limited consultations, and investigations.

But even then, health care systems and hospitals were prone to (cyber)terrorism, as shown by other COVID-19-related institutions, such as pharmaceuticals involved in developing vaccines and health care facilities involved in swab testing and contact tracing. Counter-terrorism medicine (CTM) and social behavioral science can reduce the likelihood and impact of terrorism, but cannot prevent (state-driven) cyberterrorism and actions of lone wolves and extremist factions.

## Introduction

During the coronavirus disease 2019 (COVID-19) pandemic, terrorism certainly did not disappear. The focus of the media is now mainly on the various aspects of the pandemic. Other news remains local, and international media attention is less frequent, also due to travel restrictions.

Nevertheless, both the leader of the European Union (EU) in the fight against terrorism, the Belgian Gilles de Kerchove, and the Secretary-General of the United Nations (UN), Antonio Guterres, have warned that terrorism will regain its influence.^1,2^

As already highlighted in detail in 2017, terrorist factions have previously considered the use of infectious pathogens for their activities aimed at weakening governments and instilling fear in the population, with hospitals and health care workers being a preferred target.^3,4^ For example, while the Islamic State initially called on their members to avoid COVID-19 afflicted countries, and certainly not to return from them to Islamic State-controlled territory in order not to undermine their own health system, the potential use of COVID-19 for terrorist purposes against China as a punishment for the persecution of the Uighurs was considered.^5^

Other, mostly right-wing, extremist groups also see an omen in the current pandemic to spread their ideas and recruit more allies, taking advantage of the dissatisfaction with the approach to the pandemic, the lockdown, poverty, loss of work, and closure of schools.^6^

In this special report, it is described how popular protest and civil unrest can feed extremism (both right- and left-wing) and how those, as well as terrorist factions, aim at health care workers involved in COVID-19 response (swab testing sites and contact tracing [and in near future in vaccination], epidemiologists, and governors), and at health care workers treating patients (hospitals and pharmaceuticals involved in developing drugs and vaccines). Finally, it will be discussed how severe acute respiratory syndrome coronavirus-2 (SARS-CoV-2) could be used in terrorist activities and the role of counter-terrorism medicine (CTM).

## Targeting Hospitals: From Popular Protest and Civil Unrest to the Activities of Lone Wolves

In January 2020 in Hong Kong, hotels/hospitals predestined for the reception of SARS-CoV-2 infected patients were besieged for fear of contagion of residents.^7^ In India and Cameroon, health care workers were harassed by patients and/or their relatives who did not accept the positive swab results and enforced quarantine.^8,9^ Death certificates that mentioned COVID-19 were contested. In Cameroon, the remains of COVID-19 deceased persons were exhumed by relatives to give them a “proper” burial.^9^

In several US states, there were protests against the lockdown, mainly by right-wing supporters, fueled by President Trump’s encouragement.^10^ In 2020 in Breda, the Netherlands, a swab testing facility was attacked by demonstrators who shouted “corona is a hoax!” The same occurred in Ivory Coast. In January 2021 in the Netherlands, protesters tried to get in hospitals (emergency department and maternity hospital) and torched COVID-19 testing centers during riots against the COVID curfew.^11^

Some lone wolves take it a step further. In Michigan (USA), a man was convicted of spitting on health care workers in two hospitals trying to transmit COVID-19.^12^ Also in the US, two isolated plots against health care workers have been foiled in previous months: another man from Michigan was planning to steal a helicopter and use it to fire on a hospital to “free” the COVID-19 patients.^13^ In the state of Missouri, a man was shot by the Federal Bureau of Investigation (FBI; Washington, DC USA) after resisting arrest: the man (convinced of the spread of SARS-CoV-2 by the government, led by Jews) had plans for an attack on a school with Afro-Americans, a mosque, a synagogue, and was preparing a bombing of a hospital with COVID-19 patients.^14^

## COVID-19 and Apocalyptic Thinking in Various Extremist Groups

Lone wolves are mostly inspired by the doom thinking and conspiracy theories of left-wing as well as right-wing extremism who use the COVID-19 pandemic to recruit members, proclaim their ideology, and undermine the state. Attacks on police officers, politicians, hospitals, and others are the means to further weaken police forces and health care systems that have already been overwhelmed by extra tasks due to the COVID-19 pandemic.^6,15-17^

The right-wing extremists mix racist motives, apocalyptic thinking, conspiracy theories, and anti-vaccination campaigns. This led to the return of measles because of the decline of vaccine coverage in many counties in the US and could hamper the COVID-19 response.^18^ Both right-wing extremists and Islamic State are convinced that Judgment Day is imminent and will lead to racial struggle.

The right-wing-inspired blogs blame China, Israel, and others for creating SARS-CoV-2 and asylum seekers and immigrants (especially Jews and Muslims) for wanting to spread the virus. Furthermore, they strongly proclaim that the government, with the 5G network, vaccinations, and contact tracing, wants to control the population or kill certain minorities.^6,15-17^

Another phenomenon during the COVID-19 pandemic is the misinformation that may have been sent from Russia, especially via social media. For example, the COVID-19 response in Russia is praised while fake news is spread about outcome figures in other countries, again with the stigmatization of certain population groups.^17^ Organized crime/terrorism is a real threat to hospitals.

## Hospitals as Soft Target of (Cyber)Terrorism during the Pandemic

The British National Counter Terrorism Security Office (London, United Kingdom) warned hospitals that there was a threat, especially from the Islamic State, to attack so-called “soft targets.” Lockdown measures closed the most obvious targets such a tourist attractions, public transport, and churches, mosques, and synagogues, leaving hospitals the only soft target opened 24/7.^19^

In other countries, where war is already on-going, hospitals are specially targeted by the warring factions: a maternity hospital in Kabul, Afghanistan was attacked, as well as hospitals in Tripoli and Benghazi, Libya, where the intensive care unit was destroyed to make it impossible to care for COVID-19 patients.^20,21^ Cyberterrorism was applied in COVID-19-related attacks. The constructor that built emergency hospitals for the National Health Service in the United Kingdom (London, UK) was hit by cyberterrorism.^22^ In the Czech Republic, the Brno University Hospital (Brno, Czechia), which is also the reference laboratory for COVID-19 testing, became the victim of a cyber-attack: the entire network was shut down, surgeries were cancelled, and patients were transferred to another university hospital.^23^

The people or organizations behind these attacks are unknown, but Ursula von der Leyen, president of the European Commission, criticized China for systematically attacking European health institutions.^24^ Both cyber espionage (attacks on laboratories that develop tests and vaccines) and cyber terrorism (attacks on hospitals where COVID-19 patients are cared for) are the underlying reasons for these cyber-attacks.^25^ All this forced both Microsoft (Redmond, Washington USA) and Interpol (Lyon, France) to send out a warning about the cyber threat to hospitals and other health care organizations.^26^

## When Terrorists Discover the Category A Features of SARS-CoV-2

Terrorism aims to create panic and social disruption and challenge public health services. This is exactly what COVID-19 is causing now. Category A (high-risk) agents (according to the Centers for Disease Control and Prevention [CDC; Atlanta, Georgia USA] bioterrorism agents list) include anthrax, plague, tularemia, smallpox, the hemorrhagic fever viruses, and botulinum toxin.^3^ Currently, SARS-CoV-2 is not on the list despite its unique features. All things considered, SARS-CoV-2 should be included on this list. Moreover, just like SARS and Middle Eastern Respiratory Syndrome (MERS), it is not only affecting the first targets, but it also affects the health care responders who are susceptible.^27^

Anthrax, botulinum toxin, and the Ebola virus were initially considered by the Aum Shinrikyo in Japan for bioterrorist attacks, although they eventually chose to use the nerve gas sarin instead of biological warfare.^3^ The Islamic State also considered the possibility of sending disciples to an Ebola outbreak area to deliberately expose themselves to the virus.^3^ “Mutatis mutanda,” terrorist groups could easily send members to a COVID-19 outbreak area to deliberately expose themselves to the virus, and then travel to the various targets: churches, synagogues, mosques, hospitals, demonstrations, places of pilgrimage, or security forces. It is precisely these that prove to be the most effective locations of super-spreading the pandemic.

The EU released information that Tunisian factions have tried to spread SARS-CoV-2 among the security forces.^1^ US white supremacists debated the spread of the virus on social media in February 2020, particularly targeting security forces and African Americans. Homeland Security (Washington, DC USA) revealed extremists considered leaving “saliva on door handles” at local FBI offices, spitting on elevator buttons, and spreading the virus in “non-white neighbourhoods.”^28^

## Counter-Terrorism Medicine: Intelligence, Prevention, Response, and Recovery

Counter-terrorism medicine, as a sub-specialty of disaster medicine, recently propagated by Michael Court, et al, focuses on events that include the triad of intent, violence, and health care impact through mitigation, preparedness, response, and recovery activities.^29^

Preparedness and proper response require knowledge and intelligence of those who challenge the health care system, their motivations, and their strategies which can be used in the many phases in combating the pandemic, which is mainly the scope of this manuscript. Unfortunately, there is little guidance in the literature on how to translate intelligence into prevention and health policy. Table 1 shows some practical suggestions that are useful in managing the current pandemic from a CTM perspective, including contact tracing concerns dealing with anti-science and anti-vaccination rhetoric.^30-32^ Mutatis mutandi, the recommendations for enhancing hospitals’ preparation against terrorism, listed in an earlier report, are still valid.^4^

**Table 1.** Practical Recommendations for the Management of the COVID-19 Pandemic from a Counter-Terrorism Medicine (CTM) Perspective

Topic of CTM	Recommendations
Preparedness	Provide training for hospital physicians, residents, and nurses in CBRN, in using personal protective equipment, and in terrorism risk assessment.
Prevention	Be wary of red flags in contact tracing: • Contact tracing teams should assign a terror expert (eg, an emergency physician or nurse trained in CTM) to the team to assess the clusters on terror susceptibility, especially in clusters without a clear internal index person in terror-sensitive locations: churches, synagogues, mosques, hospitals, and asylum centers. • Contract tracing apps might be prone to fraudulent modification of data in which false positives flood hospitals/triage centers, would eventually undermine government and health care systems.^30^
	Take cyberterrorism countermeasures: especially for laboratories, testing centers, and/or vaccination centers.
	Provide access barriers (against ram-raid or car bombings) at the main hospital entrance and the emergency ward entrance and at testing/vaccination centers.
Response	Anti-science, anti-lockdown, and anti-vaccination rhetoric can only be countered by profound information, open communication, and open government reaching out to the people. The lessons of the small island of Samoa in the South Pacific may inspire other countries in dealing with vaccination hesitance.^31,32^
Mitigation	• Provide a psychological debriefing team for your staff. • Limit team numbers, avoid mixing with other teams, and assign dedicated logistics staff in order to avoid large staff unavailability during outbreaks. • Provide support for employees and their families who fell ill or who are in quarantine.

Abbreviations: COVID-19, coronavirus disease 2019; CTM, counter-terrorism medicine; CBRN, chemical, biological, radiological, and nuclear.

Bavel, et al recently described the various difficulties in managing fake news and conspiracy theories, but do offer solutions for health care workers and governments to reach out to the people.^32^ Reaching out to the people positively influences citizens’ decisions to voluntarily engage in physical distancing in response to pandemic recommendations.^33^ Women governors cultivated empathy and confidence more in their COVID-19 briefings than did men governors, probably related to more adherence of recommendations and thus less COVID-19 deaths.^34^

## Discussion

The COVID-19 pandemic feeds the extremism of the extreme right and extreme left and allows the Islamic State and other militants to exploit the situation because the local governments have been weakened by the pandemic.^6^ Both the EU and the UN have warned that terrorism will regain influence.^1,2^

After Ebola and other pathogens, SARS-CoV-2 could very easily be used as a terrorist weapon, with minimum effort and maximum return on investment. Ebola has already been considered by the Islamic State and the Aum Sect in Japan, but this would mean suicide missions with probably little effect.^3^ On the other hand, terrorists using SARS-CoV-2 to spread COVID-19 would not be killed themselves, and with some luck, they would have the same effect as the Austrian bartender (ie, an outbreak throughout Europe) for the groups they are targeting (the army, mosques, synagogues, the Vatican, and political leaders [many of them are at risk: male, >70 years old, obese, and reluctant to wear mouth masks]).^35^

The World Health Organization (WHO; Geneva, Switzerland), the EU, and the national (medical) emergency plans must also focus on attacks by terrorist groups on multiple locations. The surveillance of COVID-19 using contact tracing should also include red flags for multi-site outbreaks or outbreaks in terror-sensitive places/minorities.

## Limitations

Literature on this topic is limited in medical journals. Most of the references are therefore from non-medical literature. Further research is warranted as there is little information on how to act in a proper way, as health care workers, to physical and more specifically (cyber)terrorism attacks against health care institutions/workers. Available recommendations mainly address protection against counter-(cyber)terrorism attacks in all kind of (governmental) institutions.

Another limitation is that the condition in which the world lives is constantly changing. This requires health care workers to adapt without the guidance of evidence-based roadmaps.

A third limitation is the lack of evidence about the risk health care institutions face. On the one hand, they were the only soft target still open 24/7; on the other hand, because of the lockdown measures, there was less non-essential personal traffic in the hospitals (because of suspended patient visits and limited consultations) and there was enhanced access control (temperature and mask wearing), probably reducing the likelihood and possible impact of a terrorist act.

## Conclusion

In January 2020, no one believed that hospitals in Europe would be in the line of fire of COVID-19. That same mistake should not be made again in believing that terrorist factions would not think of using this pandemic to create what they stand for: terror and panic.

Hospitals and testing centers were the only remaining “soft targets” during the lockdown, as churches, mosques, and synagogues were closed; mass gatherings (concerts and parties) were suspended; and shopping malls were closed, and thus might face a higher risk.

Counter-terrorism medicine needs more attention and academic research.
